# Plant hormone jasmonic acid reduces anxiety behavior in mice

**DOI:** 10.1038/s41598-025-95689-1

**Published:** 2025-04-03

**Authors:** Hanako Kanzaki, Shiho Suzuki, Tomotaka Tabata, Taiki Suzuki, Yoshiya Seto, Kentaro Kaneko

**Affiliations:** Department of Agricultural Chemistry, School of Agriculture, Meiji University, 1-1-1, Higashimita, Tama-ku, Kawasaki-shi, Kanagawa, 214-8571 Japan

**Keywords:** Plant hormone, Jasmonic acid, Anxiety-like behavior, Anti-stress, Nutrition, Stress and resilience

## Abstract

Anxiety disorders are a leading cause of disability worldwide and major contributors to the global disease burden. In this study, we investigated the anxiolytic-like effects of plant-derived molecules in mice. Jasmonic acid (JA), a major plant hormone, has been identified as an injury response-related hormone in higher plants. We found that the oral, intraperitoneal, and intraventricular administration of JA in mice demonstrated anxiolytic-like effects in an elevated plus maze test. Additionally, JA exhibited anxiolytic-like effects in mice undergoing open field and novel environment feeding suppression tests. In addition, we found that the anxiolytic-like effects of JA were mediated by serotonin 5-HT1A receptors and central dopamine D1 receptor systems. Our findings reveal a novel role of JA in exerting anxiolytic-like effects in animals and suggest that plant hormones, such as JA, could serve as potential compounds for treating anxiety disorders.

## Introduction

Stress is a natural human response and an essential adaptation necessary for homeostasis, performance, and survival. However, excess stress increases the likelihood of developing depression and anxiety^1^. Anxiety disorders are common psychiatric disorders of the central nervous system and are a rapidly growing health problem worldwide^2,3^. Although anxiety disorders manifest in many forms including generalized anxiety disorder, social anxiety disorder, specific phobias, and separation anxiety disorder^1^, all forms likely share the same neurological circuitry. When exposed to stress, regulating the dopaminergic reward system is essential for effectively coping with the situation. In contrast, overactivity of the central serotonergic system is associated with anxiety disorders^4^. Although certain psychological treatments have proven to be effective, pharmacotherapy remains the most widespread and effective treatment for anxiety disorders, especially in severe cases^5,6^.

The 5-HT1A receptor is considered a relevant target for the treatment of psychiatric disorders, particularly anxiety and depression^7^, and is present at both the pre- and post-synapse. When activated by systemic stimulation, somatic dendritic receptors are thought to exert anxiolytic effects^7^, reducing 5-HT release in both the cell body and terminal regions of serotonergic neurons^5^. Dopamine also regulates reward-related behaviors by activating the mesolimbic dopaminergic pathways^8^. The dopamine D1 receptor subtype mediates acute stress-induced dendritic growth in excitatory neurons of the medial prefrontal cortex^9^. Furthermore, the various drug classes used to treat depression (monoamine oxidase inhibitors, tricyclic antidepressants, and selective serotonin reuptake inhibitors) depend on the availability of monoamines (noradrenaline, dopamine, and primarily serotonin) in the brain^10,11^. Despite the availability of a wide variety of medications, approximately half of the patients seeking treatment do not respond to classical anxiolytics or antidepressants^12^. Furthermore, anxiety disorders and depression are influenced by a complex interplay of genetic, environmental, and neurobiological factors, thus making it difficult to develop universally effective treatments. Given this complexity, there is growing interest in exploring alternative pathways and determining novel mechanisms that regulate emotions. Therefore, it is necessary to identify new substances for treating anxiety and depression^13^.

Plant hormones are signaling molecules produced within plants that act as chemical messengers to regulate plant growth and mediate responses to biotic stresses^14,15^. The variety of known phytohormones, continuously growing over the years, includes abscisic acid, indole-3-acetic acid (IAA or auxin), cytokinins, gibberellic acid, ethylene, jasmonic acid (JA), and salicylic acid^15^. Plants have unique defense mechanisms against damage caused by insect feeding and physical injury^16^. For example, JA plays a central role in injury response^14,17^. When plants are exposed to injury-related stress, JA is immediately biosynthesized at the site of injury, inducing the biosynthesis of injury-response-related substances such as phytoalexins^18^. This injury response is observed not only in injured leaves but also in uninjured leaves (healthy leaves), suggesting that injury signals are translocated within the plant^19^. However, the contribution of plant hormones to animal physiological activities remains largely unknown. Indole-3-acetic acid (IAA) was discovered as a type of auxin, a plant hormone, in the 1930s when Dutch biochemists isolated it from human urine rather than from plants ^20^, suggesting that plant hormones may exist and potentially exert effects in animal systems. Although some studies have suggested potential interactions between plant-derived compounds and mammalian physiological processes^21,22^, comprehensive research on the direct effects of plant hormones on animals still remains scarce. Previous studies have mainly focused on phytoestrogens or other plant-derived bioactive compounds; however, the physiological roles of plant hormones, such as JA, in animals are still not well understood. In the present study, we hypothesized that the consumption of plant leaves induces the secretion of JA during the chewing processes in animals, and that JA may interact with animals, demonstrating physiological activity. Notably, we found that the intraperitoneal administration of JA, a plant hormone, exhibited anxiolytic-like effects in mice. In this study, we investigated whether JA mediates anxiolytic-like effects in mice using the elevated plus maze (EPM), novelty-suppressed feeding test (NSFT), and open field test (OFT).

## Materials and methods

### Materials

JA was purchased from Cayman Chemical Co. (88300; Michigan, USA). SCH23390 hydrochloride (SCH23390; R&D Systems 0925), a highly potent and selective dopamine D_1_-like receptor antagonist, and WAY100135 dihydrochloride (WAY100135, Tocris Bioscience, 1253), a potent and selective 5-HT1A receptor antagonist, were purchased from BioTechne (Minneapolis, USA). Concentrations for SCH23390 (1 μg/mouse, i.c.v.) and WAY100135 (10 mg/kg, i.p.) were determined according to previous reports^23–25^.

### Animals and housing conditions

Male C57BL/6 mice were purchased from the Japan SLC (Shizuoka, Japan). Seven-week-old mice were used for the behavioral tests. All of the mice were maintained on a 12-h light/dark cycle (lights on 7 a.m.–7 p.m.) in a temperature-controlled environment at 23 ± 1 °C with ad libitum access to water and a normal diet (MF, Oriental Yeast Co., Ltd.). The mice were administered a single oral or intraperitoneal dose of JA (100 µg or 300 µg/0.1 mL) or vehicle (saline containing 0.05% Tween 20) at 4 µL/g body weight 2 h before the behavioral tests were performed. For ICV injection studies, mice were infused with 1 μL of dimethyl sulfoxide (DMSO, vehicle) or JA (1 μg/mouse) 2 h before the mice behavioral tests were performed. All experimental procedures were conducted according to the ARRIVE guidelines.

### Cannula implantation and ICV injection

Mice were anesthetized with isoflurane and placed in a stereotaxic frame. A 26-gauge single stainless-steel guide cannula (C315GS-5-SPC, Plastics One, Roanoke, VA, USA) was implanted into the lateral ventricles (− 0.45 mm from bregma, ± 0.9 mm lateral and − 2.5 mm from the skull). The cannula was fixed to the skull using screws and dental cement. The mice were housed in groups of 6 per cage and were allowed to recover from the operation for 1 week. The placement of the guide cannula was verified histologically at the end of the experiment. Mice were infused with 1 μL of DMSO or JA (1 μg/mouse) 2 h before the mice behavioral tests were performed. SCH23390 (1 μg/mouse) was administrated 30 min before the JA injection.

### Elevated plus maze (EPM) test

The EPM test is widely used to measure anxiety-like behaviors in mice^26^. The maze (MK-10 system; Shin factory, Fukuoka, Japan) consisted of two closed arms (30 cm length × 6 cm width × 15 cm height) and two open arms (30 cm length × 6 cm width) that were elevated 50 cm above the floor. The head of the test mouse was placed at the center of the device, facing the open arm, and its behavior was observed for the next 6 min. The cumulative duration in open and closed arms, frequency of entering each arm, and the distance moved were measured using an EthoVision XT 15 system (Noldus Information Technology, Wageningen, The Netherlands/Sophia scientific, Aichi, Japan). The EPM test started at 2:00 p.m. On experimental days, the mice were injected intraperitoneally or orally with each solution: control (saline containing 0.05% Tween 20) or JA (100 µg or 300 µg/0.1 mL) 2 h before the test.

### Open field test (OFT)

The OFT has been previously used to study the neurobiological basis of anxiety in mice^27,28^. The open field (MSQ-10 system; Shin Factory, Fukuoka, Japan) consisted of four opaque walls and one floor (45 cm length × 45 cm width × 40 cm height). The test mouse was placed in the center area and its behavior was observed for the next 10 min. The cumulative duration in the center area, frequency of entering the center area, and the distance moved were measured using the EthoVision XT 15 system. The OFT started at 2:00 p.m. On the experimental days, the mice were injected intraperitoneally with each solution: control (saline containing 0.05% Tween 20) or JA (100 µg or 300 µg/0.1 mL) 2 h before the test.

### Novelty-suppressed feeding test (NSFT)

The NSFT is a behavioral test that considers an animal’s hesitation to consume highly palatable food in a novel environment as a measure of anxiety and loss of pleasure^29^. The mice were fasted the day before the test. In the OFT, one tablet of normal chow was placed in the center area, and the test mouse was placed in the corner of the border area. The cumulative duration in the central area and distance traversed were measured using the EthoVision XT 15 system. The NSFT started at 1:30 p.m. On experimental days, the mice were injected intraperitoneally with each solution: control (saline containing 0.05% Tween 20) or JA (100 µg or 300 µg/0.1 mL) 2 h before the test.

### Statistical analysis

All data were expressed as means ± SEMs. Statistical analyses were performed using a two-tailed unpaired Student’s t-test or one-way ANOVA followed by post hoc Tukey’s tests. All statistical analyses were performed using Prism version 10 (GraphPad Software, San Diego, CA, USA). Differences were considered statistically significant at a p < 0.05.

### Study approval

All procedures used to maintain and evaluate the mice followed protocols reviewed and approved by the Animal Research Committee of Meiji University (MUIACUC2022-05) (Kanagawa, Japan). This study is performed in accordance with relevant guidelines and regulations. All methods are reported in accordance with ARRIVE guidelines.

## Results

### Intraperitoneal administration of JA shows anxiolytic-like effects in mice during the EPM test

**Fig. 1:**
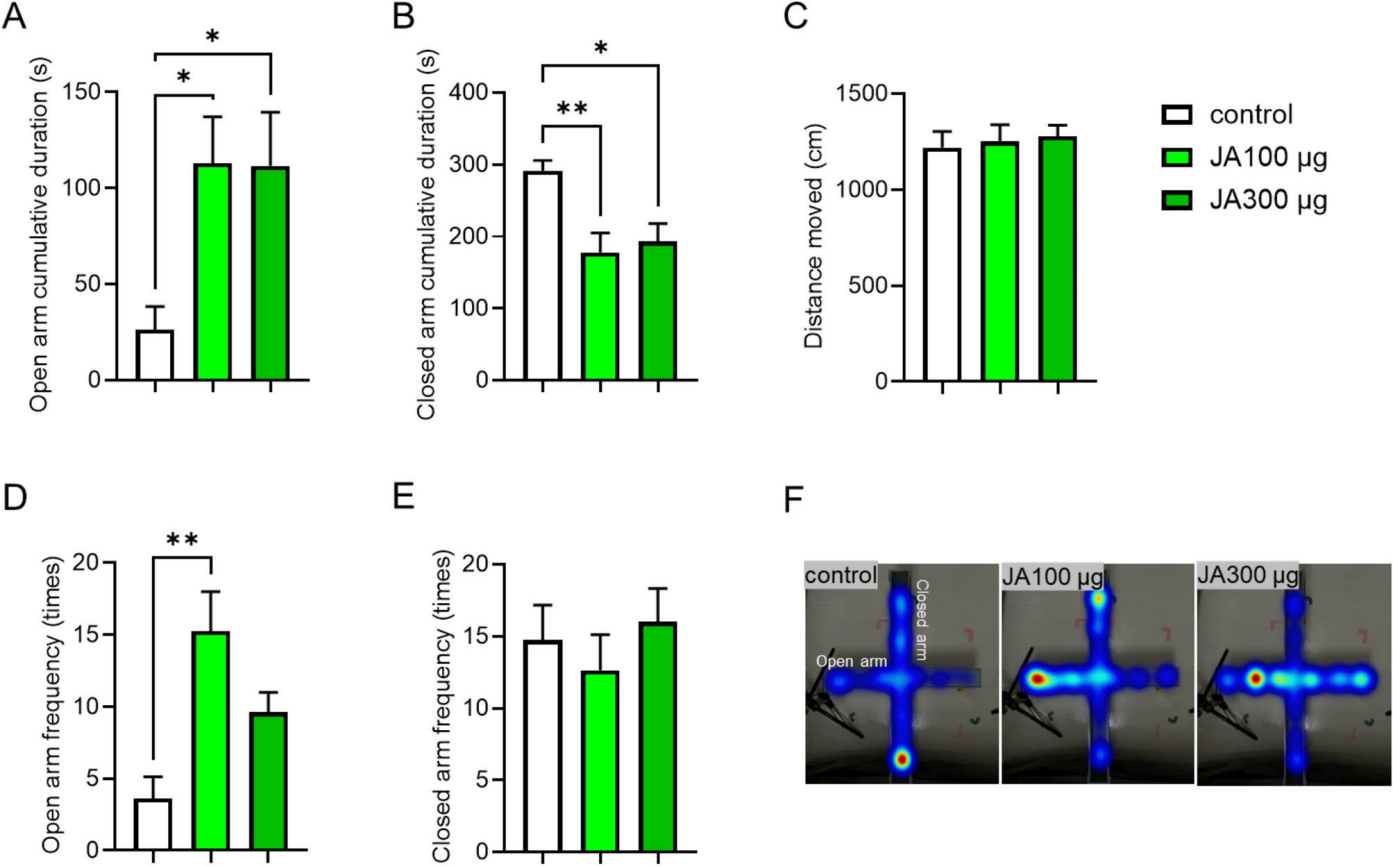
Intraperitoneal administration of JA shows anxiolytic-like effects. Open arm cumulative duration (**A**), closed arm cumulative duration (**B**), total distance moved (**C**), open arm frequency (**D**),closed arm frequency (**E**), and representative heat mapping (**F**) during a 6-min elevated plus maze test. The mice were intraperitoneally administered with vehicle (saline containing 0.05% Tween 20) or JA (100 or 300 μg/mouse) (n = 8). *p < 0.05, **p < 0.01 for one-way ANOVA followed by Tukey’s multiple comparisons tests in (**A**), (**B**), and (**D**). Data represent the means ± SEMs.

To investigate the anxiolytic-like effects of JA, we intraperitoneally administered JA (100 or 300 µg/0.1 mL) or vehicle (saline containing 0.05% Tween 20) at 4 µL/g body weight to mice and performed the EPM test. As shown in Fig. 1, acute intraperitoneal administration of JA induced a significant increase in the cumulative duration spent in the open arms (Fig. 1A), with no significant difference in the distance moved (Fig. 1C) when compared to the control group. The open arm frequency also significantly increased (Fig. 1B) in the JA-treated groups. Conversely, JA treatment caused a significant decrease in the cumulative duration in the closed arms compared with controls (Fig. 1D), with no significant difference in the closed arm frequency among the groups (Fig. 1E). Heat mapping also showed that the JA-treated groups remained longer in the open arms (Fig. 1F). These results showed that the intraperitoneal administration of JA increased the time and frequency of stay in the open arm without affecting the distance moved, suggesting that the intraperitoneal administration of JA has anxiolytic-like effects in mice.

### JA shows anxiolytic-like effects during the OFT and NSFT

**Fig. 2:**
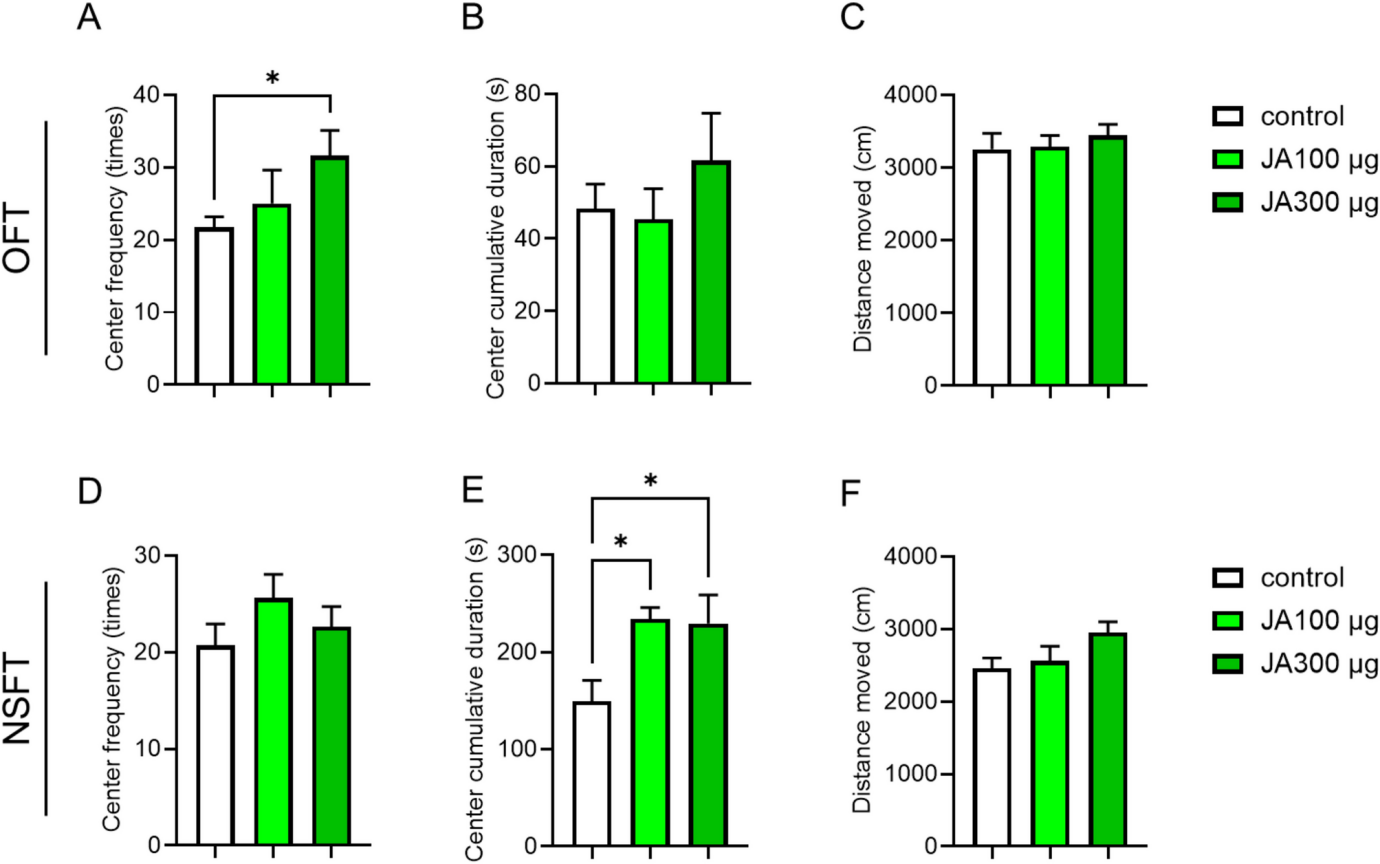
JA treatment shows anxiolytic-like behavior in OFT and NSFT. Center frequency (**A**), center cumulative duration (**B**), and total distance moved (**C**) during a 10-min open field test. Center frequency (**D**), center cumulative duration (**E**), and total distance moved (**F**) during the 10-min novelty-suppressed feeding test. The mice were intraperitoneally administered with vehicle (saline containing 0.05% Tween 20) or JA (100 or 300 μg/mouse) (n = 8 for OFT, n = 8–11 for NSFT). *p < 0.05 for one-way ANOVA followed by Tukey’s multiple comparisons tests in (**A**) and (**E**). Data represent the means ± SEMs.

To further study the potential anxiolytic-like effects of JA, we performed another anxiety-related behavioral assessment. In addition to EPM, the OFT is widely used to study the neurobiological basis of anxiety and screen for novel anxiolytic compounds. The OFT was performed 2 h after the intraperitoneal administration of JA; on examining the three groups (control, JA 100 μg, and JA 300 μg), the frequency of entering the center was significantly increased in the JA-treated groups (Fig. 2A), with no significant difference in the distance moved (Fig. 2C). The cumulative duration spent in the center area slightly increased in the JA 300 μg group compared with the control (Fig. 2B).

Next, we performed the NSFT, which is used to evaluate the activity of anxiolytic- or antidepressant-like compounds in mice. The mice were fasted the day before the test. The NSFT was performed 2 h after the intraperitoneal administration of JA; on examining the three groups (control, JA 100 μg and JA300 μg), the cumulative duration spent in the center significantly increased in the JA-treated groups (Fig. 2E), with no significant difference in the distance moved (Fig. 2F). The frequency of entering the center was slightly increased in the JA-treated groups compared to that in the control (Fig. 2D). Thus, the intraperitoneal administration of JA exhibited anxiolytic-like effects based on results from multiple behavioral paradigms that evaluated anxiety in mice.

### Oral and central administration of JA exhibits anxiolytic-like effects

**Fig. 3:**
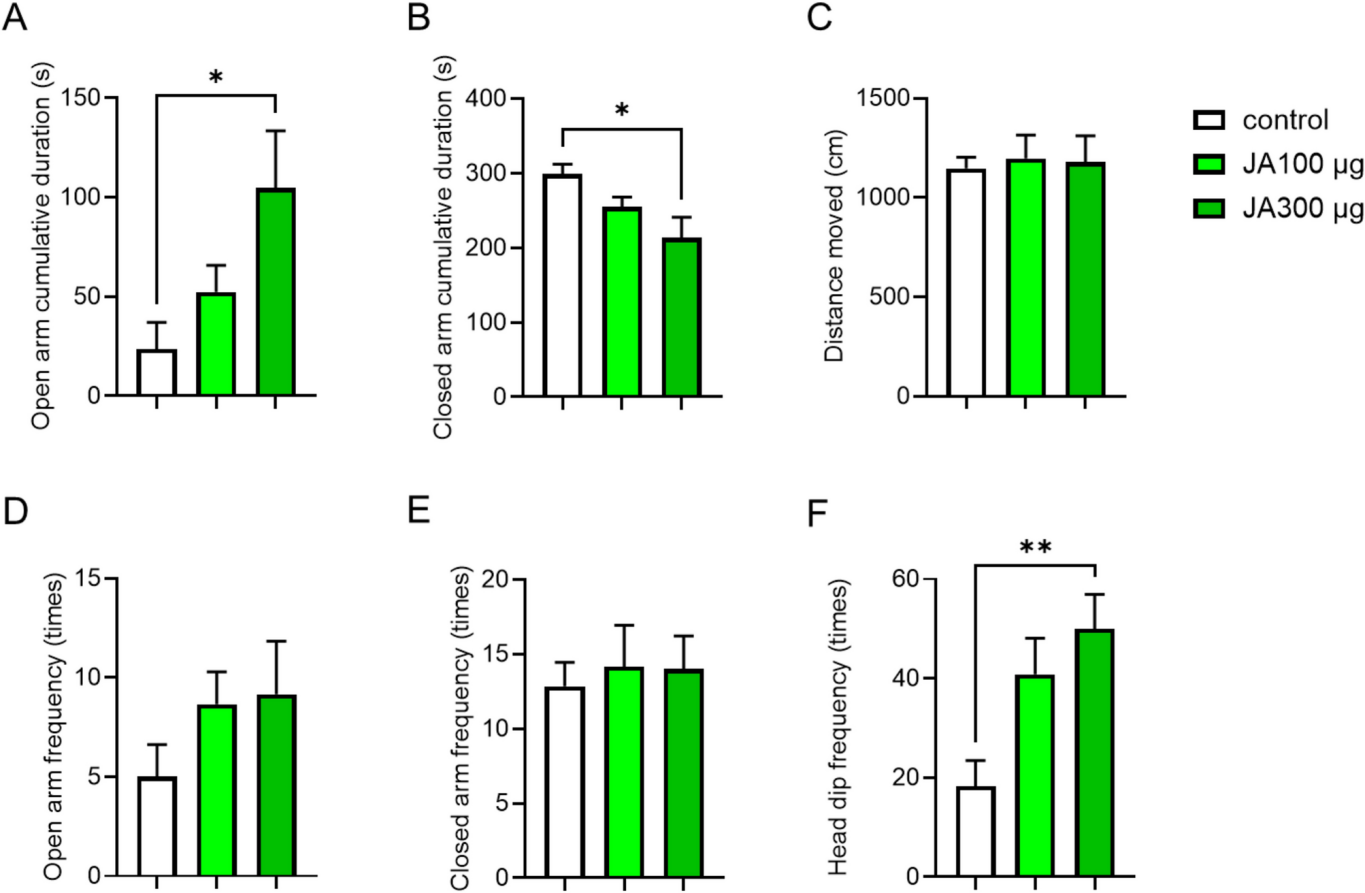
Oral administration of JA has anxiolytic-like effects in mice. Open arm cumulative duration (**A**), closed arm cumulative duration (**B**), total distance moved (**C**), open arm frequency (**D**), closed arm frequency (**E**), and head dip frequency (**F**) during a 6-min elevated plus maze test. The mice were orally administered with vehicle (saline containing 0.05% Tween 20) or JA (100 or 300 μg/mouse) (n = 6). *p < 0.05, **p < 0.01 for one-way ANOVA followed by Tukey’s multiple comparisons tests in (**A**), (**B**), and (**F**). Data represent the means ± SEMs.

To determine whether orally administered JA exhibited anxiolytic-like effects similar to those of intraperitoneal administration, JA was orally administered to mice. Oral JA treatment significantly increased the open arm cumulative duration (Fig. 3A), with no significant difference in the distance moved (Fig. 3C) during the EPM test. The closed arm cumulative duration also significantly decreased (Fig. 3B), while the open arm frequency increased (Fig. 3D) in JA-treated mice, whereas no significant difference in the closed arm frequency was observed (Fig. 3E). The head dip frequency was defined as the number of times the mouse looked down in the open arm area, which was significantly increased in the JA group (300 μg/mouse) compared with the control (Fig. 3F). These results suggest that the oral administration of JA has an anxiolytic-like effect in mice.

**Fig. 4:**
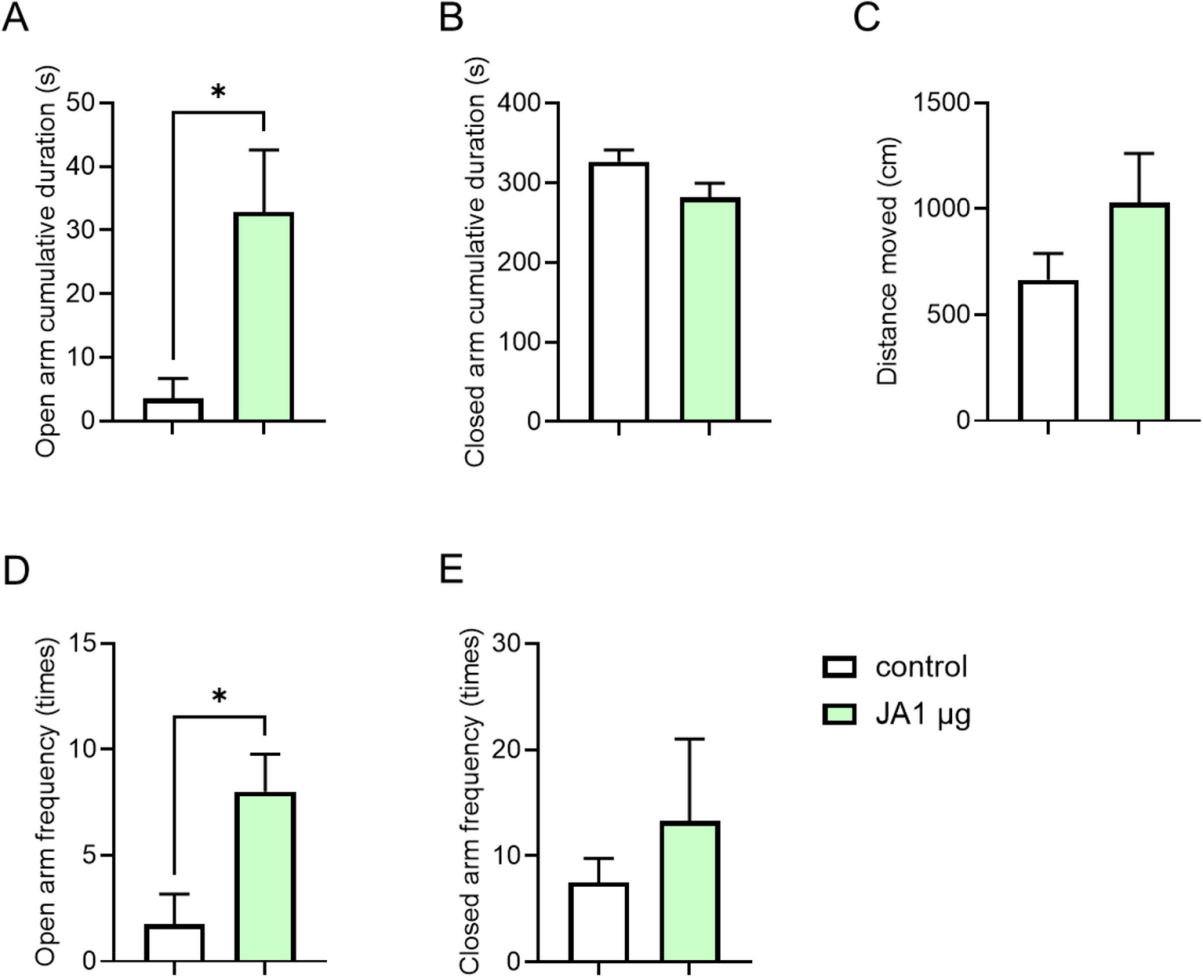
Central administration of JA shows anxiolytic-like activity in mice. Open arm cumulative duration (**A**), closed arm cumulative duration (**B**), total distance moved (**C**), open arm frequency (**D**), and closed arm frequency (**E**) during a 6-min elevated plus maze test. The mice were centrally administered with vehicle (DMSO) or JA (1 μg/brain/mouse) (n = 4). *p < 0.05, t-tests in (**A**) and (**D**). Data represent the means ± SEMs.

As many studies have demonstrated that the central nervous system plays a crucial role in anxiety behavior, we tested whether JA showed anxiolytic-like effects after ICV treatment. As shown in Fig. 4, the ICV injection of JA significantly increased the open arm cumulative duration in the JA-treated group (Fig. 4A), with no significant difference observed in the distance moved (Fig. 4C). Central administration of JA decreased the closed arm cumulative duration (Fig. 4B) and significantly increased the open arm frequency (Fig. 4D); however, there was no significant difference in the closed arm frequency between the groups (Fig. 4E). These results showed that the ICV injection of JA resulted in anxiolytic-like effects in mice. Overall, these findings suggest that plant-derived JA exerts anxiolytic-like effects in mice via intraperitoneal, oral, and ICV injections.

### Involvement of the dopamine D1 receptor and serotonin 5-HT1A receptor system in the anxiolytic-like effect of JA

Serotonin and dopamine are key neurotransmitters involved in anxiety neurobiology. Therefore, in order to investigate the mechanism underlying the anxiolytic-like effects of JA, we focused on the dopamine and serotonin systems.

**Fig. 5:**
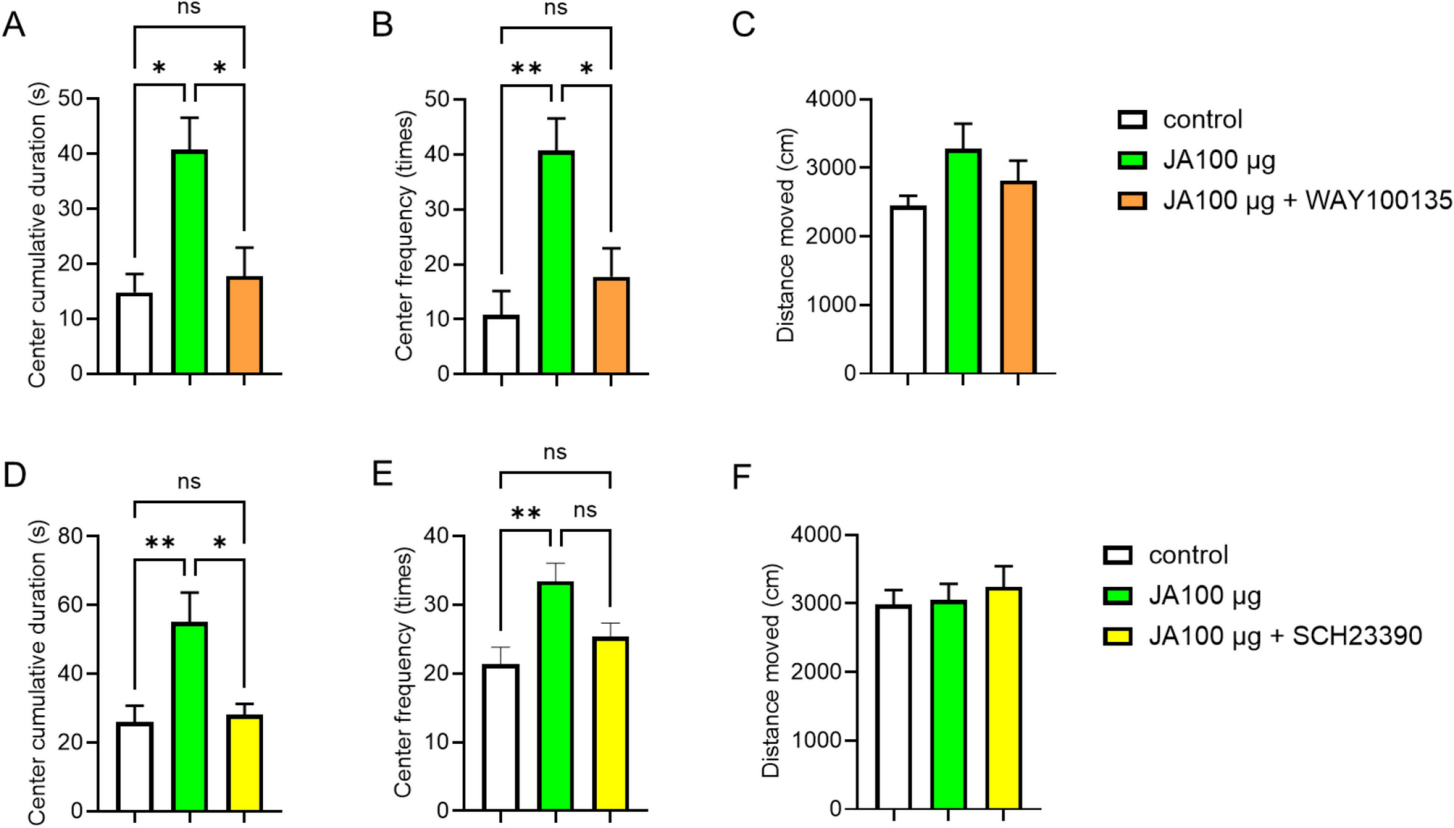
Blockade of serotonin 5-HT1A receptor and central dopamine D1 receptor systems abolished the JA-induced anxiety-like behavior. (**A**–**C**) The mice were intraperitoneally administered with vehicle (saline containing 0.05% Tween 20) or WAY100135 (250 μg/mouse). Then, 30 min after antagonist injection, the mice were intraperitoneally administered with vehicle or JA (100 μg/mouse) (n = 4). The center cumulative durations (**A**), center frequency (**B**), and total distance moved (**C**) during the 10-min open field test. (**D**–**F**) The mice were centrally administered with vehicle (DMSO) or SCH23390 (1 μg/brain/mouse). Then, 30 min after antagonist injection, the mice were intraperitoneally administered with vehicle or JA (100 μg/mouse) (n = 6). The center cumulative durations (**D**), center frequency (**E**), and total distance moved (**F**) during a 10-min open field test. *p < 0.05, **p < 0.01 for one-way-ANOVA followed by Turkey’s multiple comparisons tests in (**A**), (**B**), (**D**), and (**E**). Data represent the means ± SEMs.

WAY100135, a highly selective 5-HT1A receptor antagonist (Fig. S1), was administered intraperitoneally, and JA was administered intraperitoneally 30 min after the antagonist injection. Although the cumulative duration spent in the center and the frequency of entering the center increased in JA-treated mice compared to those in the control group (Fig. 5A and B), WAY100135 significantly blocked these JA-induced increases. Furthermore, there was no significant difference in distance moved between the groups (Fig. 5C). These results suggest that 5-HT1A receptors are involved in the anxiolytic-like effects of JA.

Next, SCH23390, a specific D_1_ receptor antagonist (Fig. S2), was administered centrally, and 30 min after the antagonist injection, JA was administered intraperitoneally. The OFT was performed 2 h after the JA injection. The cumulative duration spent in the center and the frequency of entering the center increased in the JA-treated group compared to those in the control group (Fig. 5D and E). The JA-induced increase in the cumulative duration spent in the center was significantly blocked by the central administration of SCH23390. In addition, there was no difference in the distance moved between the groups (Fig. 5F). These findings suggest that in addition to the involvement of the serotonin 5-HT1A receptor, JA regulates anxiolytic-like effects through the dopamine D_1_ receptor system in the brain. Therefore, we uncovered a previously unidentified anxiolytic-like effect of the plant-derived hormone JA in mice and revealed that this effect might involve the central dopamine and serotonin systems.

## Discussion

In this study, we aimed to elucidate the role of JA, a plant hormone, in animals by administering it to mice and revealed for the first time that JA exhibits an anxiolytic-like effect. JA is an important molecule in the regulation of many physiological processes during plant growth and development, especially in the mediation of plant responses to biotic and abiotic stresses^14^. When plants are attacked by insects, they release not only JA but also methyl jasmonate, a volatile compound. Methyl jasmonate is a form of JA used as an indicator to attract predators and parasites of herbivorous insects. Plants exposed to these volatile compounds induce the expression of defense-responsive genes and increase their resistance^30^. Methyl jasmonate has been isolated from jasmine oil and is a component in the scent of jasmine^31,32^. The volatile nature of methyl jasmonate has led to the discovery of its role as a signal in plant cellular responses, plant–herbivore interactions, and plant-plant interactions^32^. Previous studies have reported that the aroma components of coffee and tea, which include methyl jasmonate, exhibit anti-stress effects^33^. However, these volatile components are not intended for oral consumption, and no oral effects were expected^33^. In particular, a previous study reported that the intraperitoneal administration of methyl jasmonate, a volatile component, did not show a significant difference in the open-arm residence time during EPM tests at any concentration^33^. The authors also reported that the intraperitoneal administration of methyl jasmonate exhibited anxiolytic-like effects in mice subjected to OFTs at concentrations ranging from 200 to 400 mg/kg, considered as “at high doses,” when compared to the anxiolytic-like effects of JA, at 100 μg (4 mg/kg) or 300 μg (12 mg/kg), which were effective in this study^33^. Previous studies have primarily focused on the volatile methyl jasmonate, whereas the physiological functions of the non-volatile JA, the focus of this study, were not elucidated. This study is the first to show that oral administration of JA induces anxiolytic-like effects, suggesting an interaction between JA and animals. Since few reports have demonstrated the physiological functions of orally consumed plant hormones, it is necessary to elucidate the underlying mechanisms of their effects in animals. The physiological and nutritional roles of JA in mice require further evaluation.

Anxiety is an unpleasant and elusive feeling associated with inquietude behavior. Approximately one-eighth of the world’s population suffers from anxiety, which is one of the most common psychiatric disorders^34^. Common anti-anxiety medications have been developed using anxiety-like behavior tests, such as the EPM, OFT, and NSFT. The EPM is a standardized test for evaluating anxiety-like behavior in laboratory animals^26^, while the OFT measures locomotor activity and anxiety in rodents^27,28^. Similar to the OFT, the NSFT is often used to measure anxiolytic-like behaviors^29^. Thus, these tests are commonly used to evaluate the anxiolytic effects of pharmaceutical compounds. Through these tests, we showed that oral, intraperitoneal, and central administration of JA had anxiolytic-like effects in mice. These findings indicate that plant hormones, such as JA, are novel targets for the development of anxiolytic compounds.

Neurotransmitters are involved in the modulation of anxiety^35^. In particular, the serotonin and dopamine systems play central roles in regulating emotional behaviors. In this study, we found that dopamine D1 and serotonin 5-HT1A receptor antagonists abolished JA-induced anxiolytic-like effects. Although the JA-induced anxiolytic-like effect is mediated by the serotonin 5-HT1A and dopamine D2 receptor systems, the underlying mechanisms remain unexplored. Therefore, elucidating whether JA reaches the brain and induces anxiolytic-like effects in the central nervous system and the specific mechanisms underlying the roles of JA treatments in neurotransmitter systems, which are associated with emotional behaviors, are important topics for future research.

Dietary fibers and proteins are well-known plant-based resources; however, in this study, we discovered new plant-derived molecules that interact with animals. Because plants cannot move on their own, they secrete hormones to overcome environmental stresses, such as nutrient starvation, drought, temperature diseases, and insect damage, and maintain their physiological functions^36^. In the present study, we hypothesized that vegetable processing, such as cooking or chewing, in humans may induce the secretion of plant hormones that are absorbed and interact with animals. Considering that JA is secreted in response to insect damage or injury ^16^, we tested whether JA secretion increased during vegetable processing. We previously reported that JA accumulates in systemic tissues in response to mechanical wound stress in tobacco plants (Nicotiana tabacum), based on kinetic studies of the accumulation of JA and JA-related compounds^19^. When commercially available spinach was chopped with kitchen scissors and polytrons, we also observed that the endogenous amount of JA significantly increased in the wounded group (90 min after wounding) compared to the unwounded control group. These results suggest that JA secretion is promoted during cooking and chewing processes. If we can elucidate the mechanisms by which plant hormones control biological and physiological functions, plant hormones may provide new opportunities to discover novel approaches that can improve and prevent diseases.

To address and support global sustainable development goals, we should consider reducing the intake of animal products while increasing the use of plant-based products from diverse sources. If unused plant resources, such as plant hormones, can influence appetite, motivation, cognitive function, and sleep quality, they could pave the way for the development of new functional foods derived from plants. Furthermore, we aim to promote a new aspect of dietary education that emphasizes the stress-reducing benefits of chewing vegetables thoroughly and consuming them. Collectively, our findings demonstrate the potential of JA as an anxiolytic-like compound and provide insights into the potential translational value of plant hormones.

## Supplementary Information

Supplementary Information.

## Data Availability

Data is provided within the manuscript or supplementary information files. The datasets used and/or analyzed in the current study are available from the corresponding author upon reasonable request.

## Abbreviations

JA: Jasmonic acid
ANOVA: Analysis of variance
ICV: Intracerebroventricular
SEM: Standard error of the mean
OFT: Open field test
EPM: Elevated plus maze
NSFT: Novelty-suppressed feeding test
