# Supplementary material for: Plant hormone jasmonic acid reduces anxiety behavior in mice
Source: Sci Rep. 2025 Apr 3;15:11424. doi: 10.1038/s41598-025-95689-1 (PMC11968907; doi:10.1038/s41598-025-95689-1)
Supplement: Supplementary file 1 — Supplementary Information. [file 41598_2025_95689_MOESM1_ESM.pptx]

## Slide 1
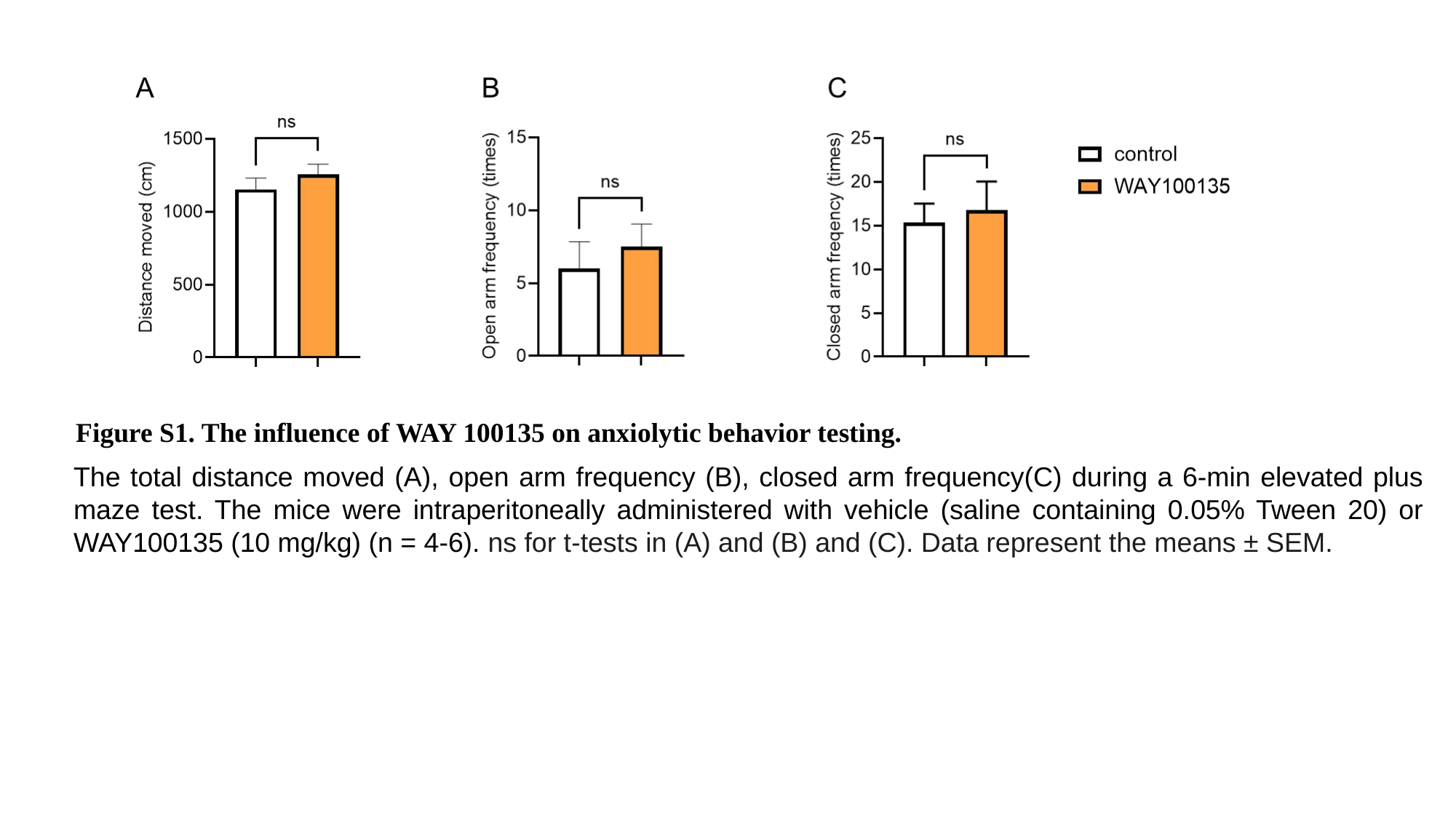

Figure S1. The influence of WAY 100135 on anxiolytic behavior testing.
The total distance moved (A), open arm frequency (B), closed arm frequency(C) during a 6-min elevated plus maze test. The mice were intraperitoneally administered with vehicle (saline containing 0.05% Tween 20) or WAY100135 (10 mg/kg) (n = 4-6). ns for t-tests in (A) and (B) and (C). Data represent the means ± SEM.

## Slide 2
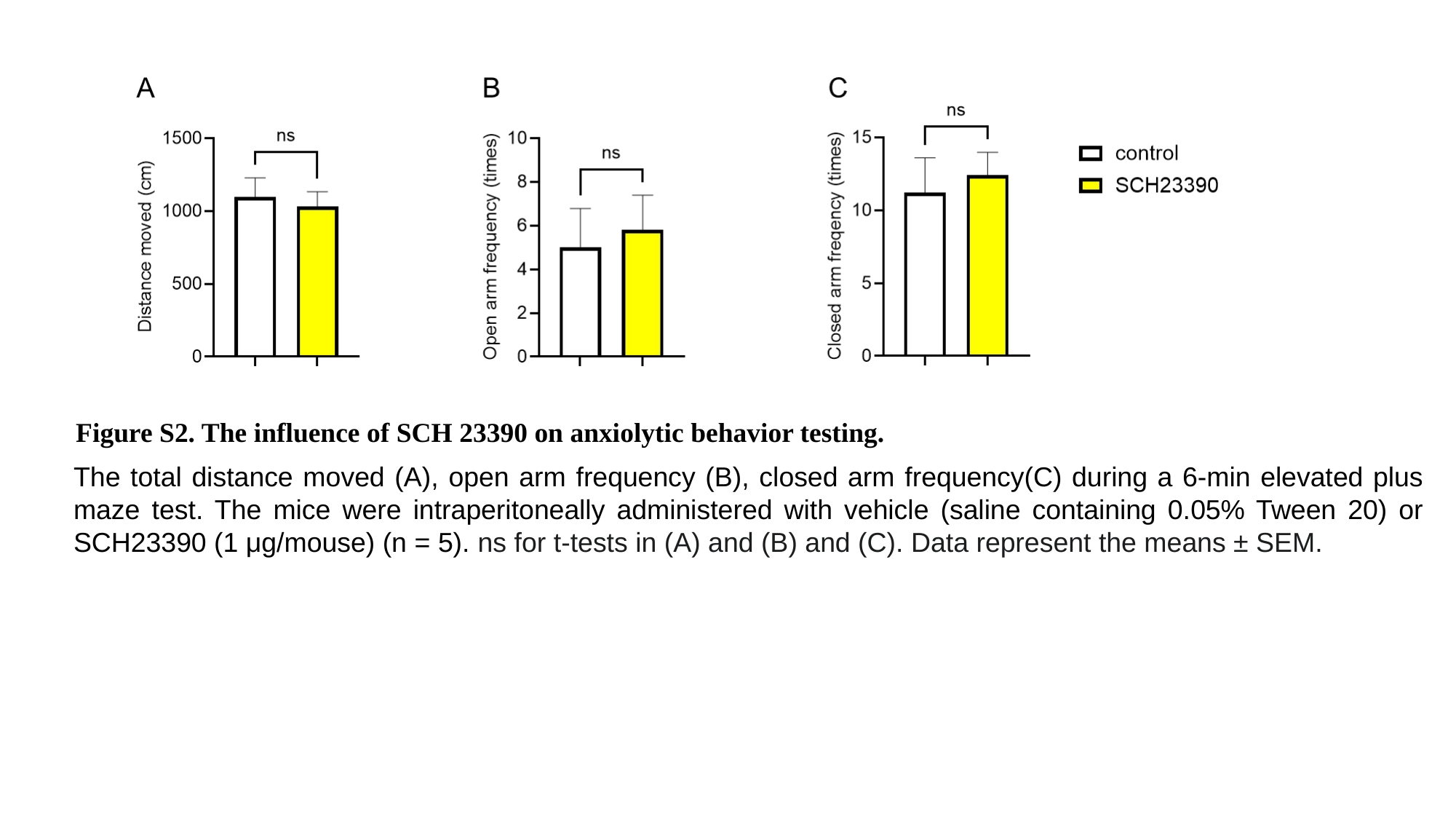

Figure S2. The influence of SCH 23390 on anxiolytic behavior testing.
The total distance moved (A), open arm frequency (B), closed arm frequency(C) during a 6-min elevated plus maze test. The mice were intraperitoneally administered with vehicle (saline containing 0.05% Tween 20) or SCH23390 (1 μg/mouse) (n = 5). ns for t-tests in (A) and (B) and (C). Data represent the means ± SEM.
